# Effects of a valved infant-bottle with ergonomic teat on the coordination of sucking, swallowing, and respiration in late-preterm infants. The Safe Oral Feeding randomized Trial

**DOI:** 10.3389/fped.2024.1309923

**Published:** 2024-01-11

**Authors:** Francesco Cresi, Elena Maggiora, Martina Capitanio, Cecilia Bovio, Federica Borla, Sara Cosimi, Desirèe Enrietti, Francesca Faggiano, Sara Loro, Serena Maria Rovei, Federica Runfola, Mariangela Scrufari, Michela Vigna Taglianti, Federica Vignali, Chiara Peila, Alessandra Coscia

**Affiliations:** ^1^ Neonatal Intensive Care Unit, Department of Public Health and Pediatrics, University of Turin, Turin, Italy; ^2^ Department of Obsterics and Gynecology, Città Della Salute e Della Scienza di Torino, Turin, Italy

**Keywords:** oral feeding, bottle, cardiorespiratory events, sucking behaviour, feeding behaviour, feeding skills

## Abstract

**Introduction:**

Breastfeeding naturally enables the coordination of sucking, swallowing, and respiration patterns for safe feeding. When breastfeeding is not possible a feeding device that releases milk in response to intra-oral vacuum could potentially offer improved coordination of sucking, swallowing, and breathing patterns compared to conventional devices. The aim of the study is to evaluate the effect of a valved infant-bottle with an ergonomic teat compared to a standard infant-bottle.

**Methods:**

This unblinded randomized controlled trial focused on late preterm infants fed by bottle for at least three meals over the day, admitted to the Neonatal Unit of Sant’Anna Hospital (Turin, Italy). Infants were randomized to be fed with a valved infant-bottle with an ergonomic teat (B-EXP arm) or with a standard infant-bottle (B-STD arm). Monitoring included a simultaneous synchronized recording of sucking, swallowing and respiration. The main outcome was the swallowing/breathing ratio.

**Results:**

Forty infants (20 B-EXP arm; 20 B-STD arm) with a median gestational age of 35.0 weeks (IQR 35.0–36.0 weeks) completed the study. Four infants were censored for the presence of artifacts in the polygraphic traces. The median swallowing/breathing ratio was 1.11 (1.03–1.23) in the B-EXP arm and 1.75 (1.21–2.06) in the B-STD (*p* = .003). A lower frequency of swallowing events during the inspiratory phase of breathing was observed in B-EXP arm compared with B-STD arm (*p* = 0.013).

**Discussion:**

The valved infant-bottle with an ergonomic teat improves the coordination of sucking-swallowing-respiration and limits the risk of inhalation reducing the frequency of swallowing during the inspiratory phase.

## Introduction

Sucking, swallowing and breathing coordination in newborns, especially preterm infants, is not fully developed at birth, leading to feeding challenges (1, 2). This is due to their struggle to synchronize sucking, swallowing, and breathing, potentially causing complications like oxygen desaturation or inhalation of milk (3).

In full term infants breastfeeding promotes sucking, swallowing and respiration coordination and maintains steady oxygen levels, whereas bottle-feeding can disrupt this balance (4). Healthy term breastfed infants typically follow a 1:1:1 sucking-swallowing-breathing pattern, whereas bottle-fed infants might have irregular patterns, risking breathing pauses (5, 6).

Late-preterm babies face even greater challenges due to their immature respiratory centers and coordination. Additionally, infants smaller for their gestational age can display delayed sucking development (7).

Despite its benefits in feeding coordination, breastfeeding is highly demanding for premature infants who often struggle to achieve exclusive breastfeeding. Moreover, exclusive breastfeeding is not always available in the first days of life.

Thus, offering feeding devices that mimic breastfeeding is crucial to promote proper coordination and oxygen saturation (4).

Research on term babies underscores the importance of mouth negative pressure for milk extraction and proper breathing (8). Our hypothesis was that an enhanced feeding system requiring additional negative pressure for milk extraction could provide better coordination and stability than conventional devices.

Aim of this study was to evaluate the effects of a feeding system based on a valved infant-bottle with an ergonomic teat compared with a standard infant-bottle on the coordination of the sucking-swallowing-breathing sequence in late preterm infants.

## Material and methods

This unblinded randomized controlled trial (RCT) was conducted in the Neonatal Unit of the Sant’Anna Hospital, “Città della Salute e della Scienza di Torino”, Turin, Italy. The study protocol was reviewed and approved by the Institutional Review Board and the local Ethics Committee (approval number 0128104). The CONSORT guidelines (9) were adopted for this RCT and it was registered on ClinicalTrials.gov (ID: NCT04400175).

The intervention was the randomization to be fed by a valved infant-bottle with an ergonomic teat (B-EXP arm) or to be fed by a standard infant-bottle (B-STD arm).

### Population of the study

The population of the study consisted of late preterm infants admitted to the Neonatal Unit of Sant’Anna Hospital and consecutively enrolled between December 2021 and May 2023. These infants met specific criteria: 34^+0^–36^+6^ weeks’ gestation at birth, exclusive oral feeding, bottle-fed for at least three meals over a day, parental written consent to participate into the study. Trust interpreter and link worker services were used to support involvement of participants whose first language was not Italian.

Informed written consent forms was prepared according to the Declaration of Helsinki and was collected and stored by the principal investigator. All investigators conducted the study according to the rules of Good Clinical Practice (CPMP/ICH/135/1995 and decree law of the Italian Ministry of Health).

Infants with exclusive breastfeeding, and/or congenital anomalies, perinatal asphyxia, need of respiratory support, neurological issues, genetic syndromes, infections, and metabolic diseases were excluded from the study.

### Study design

Eligible infants were allocated to the “B-EXP” or “B-STD” arm by block randomization. The randomization list was provided by a software.

Enrolled infants were monitored for the entire duration of a meal given by the feeding bottle assigned at randomization (study-meal) at 24–72 h of life. A 3 meals training was performed by every infant using the feeding bottle assigned by randomization.

The study-meal was administered by a trained caregiver (a parent or a nurse). During the study-meal the infant was wrapped in the caregivers’ arms in a semi-elevated side-lying position. Infants were fed with defined quantities (10 ml/kg) of maternal or donated human milk.

All mothers were offered support designed to encourage breastfeeding, according to the protocol in use in our unit. When breastfeeding was not possible, infants continued to use the bottle assigned at randomization until discharge.

### Feeding devices

Infants in the B-EXP arm were fed using a feeding system (Chicco PERFECT 5 Biofunctional feeding bottle with the intuit-flow system Physio teat and Equilibrium membrane, Artsana, Italy) composed of a slow-flow ergonomic silicon teat, a 150 ml polypropylene bottle equipped with a ventilation silicon valve on the bottom.

The valve is designed to allow air to enter the bottle when the infant exerts negative pressure. This mechanism serves a dual purpose: preventing milk from escaping from the teat when the infant is not ready to swallow and preventing the creation of negative pressure inside the bottle as the feed is consumed.

The teat was designed to mimic the shape of the mother's nipple as reshaped by the infant's sucking, elongated and widened to encourage a sucking action that closely approximates the natural peristaltic motion of the tongue. Its broad base is designed to facilitate a secure latch by the infant. The nipple is made from soft, flexible silicone to accommodate the movements of the baby's tongue.

Infants in the B-STD arm were fed using a classic disposable feeding device in use in our unit, composed of a 150 ml polypropylene bottle and a silicon teat.

All components of both feeding devices were Bisphenol-A free.

**Figure 1:**
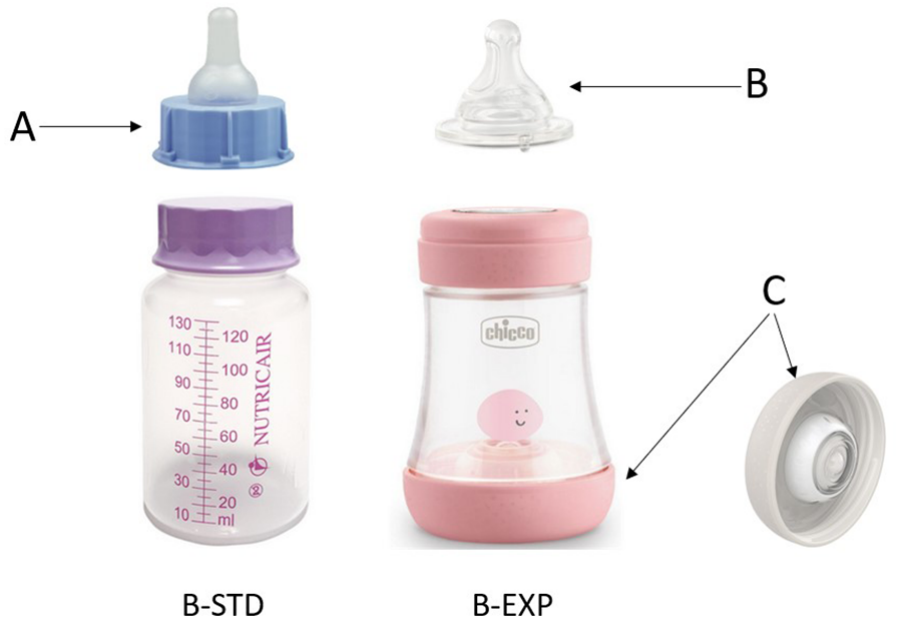
Standard and experimental feeding devices. B-EXP, valved infant-bottle with ergonomic teat; B-STD, standard infant-bottle; (**A**) standard silicone teat equipped with a connecting ring at the base to allow free air entry; (**B**) silicone teat designed to promote optimal adherence to the neonate's mouth; (**C**) diaphragm valve engineered to facilitate air entry when subjected to negative pressure.

The B-EXP and B-STD power devices are illustrated in Figure 1A.

### Instrumental monitoring

The monitoring procedure consisted of a simultaneous non-invasive recording of sucking, swallowing and cardiorespiratory activity for the entire duration of the meal.

**Figure 2:**
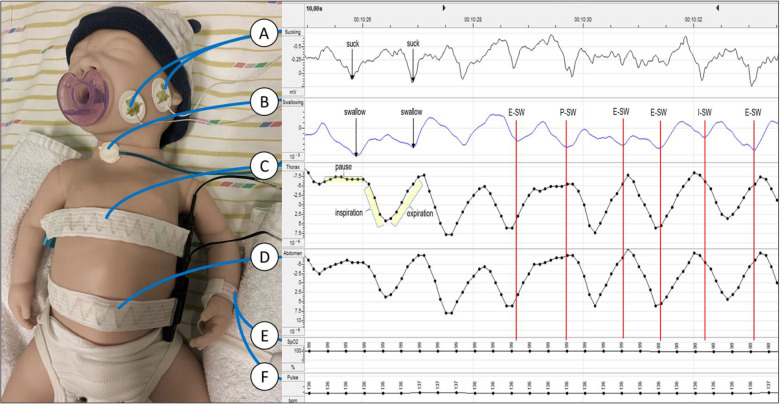
Data acquisition and interpretation of traces. (**A**) Couple of skin electrodes placed on the masseter to detect sucking activity; (**B**) graseby capsule placed on the lower anterior medial part of the neck to record swallowing events; (**C**) thoracic band for respiratory inductance plethysmography; (**D**) abdominal band for respiratory inductance plethysmography (**E**) pulse-oximeter sensor to record blood oxygen saturation; (**F**) pulse-oximeter sensor to record heart rate; E-Sw, swallowing event occurred during expiration; I-Sw, swallowing event occurred during inspiration; P-Sw, swallowing event occurred during respiratory pause.

Data monitoring was performed by Embletta® MPR device (Natus, WI, USA) connected with a laptop computer. (Figure 2).

Sucking movements were studied through skin electrodes placed on the masseter muscle.

Swallows were studied through a Graseby capsule (Graseby Medical, UK) placed on the lower anterior medial part of the neck. Graseby capsule is a little balloon filled with air, sensitive to pressure variations, connected to a pressure transductor.

Respiratory activity was monitored by two bands for respiratory inductance plethysmography positioned on the chest and on the abdomen.

Heart frequency and blood oxygen saturation were measured through a pulse-oximeter with a sensor placed on the wrist or foot.

Data recorded were visually analyzed by two trained operators blind to arm allocation.

The following features were evaluated:

• A suck event was defined as a drop of impedance to 50% of the basal value followed by a recovery of the impedance baseline values measured between the two skin electrodes placed on the masseter.
• A swallow event was defined as a drop of pressure to 50% of the basal value followed by a recovery of the pressure baseline values measured by the Graseby capsule.
• A breathing event was defined as a drop of impedance to 50% of the basal value followed by a recovery of the impedance baseline values measured by the thoracic band.
• Inspiration was defined as the portion of breathing between the maximum and minimum impedance value recording during the breathing event.
• Expiration was defined as the portion of breathing between the minimum and maximum impedance value recording during the breathing event.
• Respiratory pauses were defined as the time occurred between two breathing events.
• Apnoea event was defined as an arrest of the breathing acts for at least 20 s or arrest of the breathing acts for a period of at least 5 s associated with bradycardia (heart rate <80 bpm) and/or desaturation (SpO2) <80% (10).

Swallow runs were defined as at least three swallows with inter-swallow intervals of not more than 2 s (11). Sucking interruptions longer than 2 s were considered breaks and were excluded from the analysis.

The effective extraction time was calculated as the difference, in seconds, between the meal duration and the total duration of sucking interruptions.

Finally, swallowing events were classified by the respiratory phase in which they occurred and were categorized into three classes: swallowing events during inspiration (I-Sw), swallowing events during expiration (E-Sw), and swallowing events during respiratory pause (P-Sw). (Figure 2).

The primary outcome was the level of coordination of the sucking, swallowing, and breathing sequence defined as swallowing/breathing ratio.

Secondary outcomes were the median volume of administered meal, the median meal duration, the median duration of the inter-swallows intervals, the median milk flow, the median frequency of sucking, swallowing, and breathing events, the median sucking/swallowing and sucking/breathing ratio, the median apnoea frequency, the median blood oxygen saturation and heart rate, and the prevalence of breastfed infants at discharge.

### Data analysis and statistics

Statistical analysis was carried out using the STATISTICA software package for Windows (StatSoft, Inc., Tulsa, Oklahoma, USA). Normally distributed data were tested with the *Kolmogorov–Smirnov* test. Data were expressed as statistical mean and standard deviation (SD) or as median and interquartile range depending on the relative distribution. The differences between the polygraphic data were evaluated with the Student's *T* test or with the Mann Whitney test depending on the distribution. Fisher test was used for comparison between categorical data.

Specific data regarding the swallowing/respiration ratio in bottle-fed late preterm infants in the first days of life are lacking in the literature. Considering a mean swallow/respiration ratio of 1.5 and a variance of 0.5, obtained from a pilot study performed in our center prior to this study, a minimum sample size of 38 infants with a 1:1 group allocation was estimated to achieve 80% power to detect a 30% difference on the primary outcome with a statistical significance level set at 0.05.

## Results

During the study period 66 infants were eligible. Among them 40 infants were enrolled into the study, 20 (50%) infants in B-EXP and 20 (50%) in B-STD arm. The median gestational age at birth was 35.0 weeks (IQR, 35.0–36.0 weeks) and the median birth weight was 2,540 g (IQR, 2,390–2,600 g). Twenty infants (55.5%) were female. All infants involved in the study were given breast milk or human bank milk according to the standard care procedures of the Center before their inclusion in the study. Additionally, none of these infants needed respiratory support before their participation in the study.

**Figure 3:**
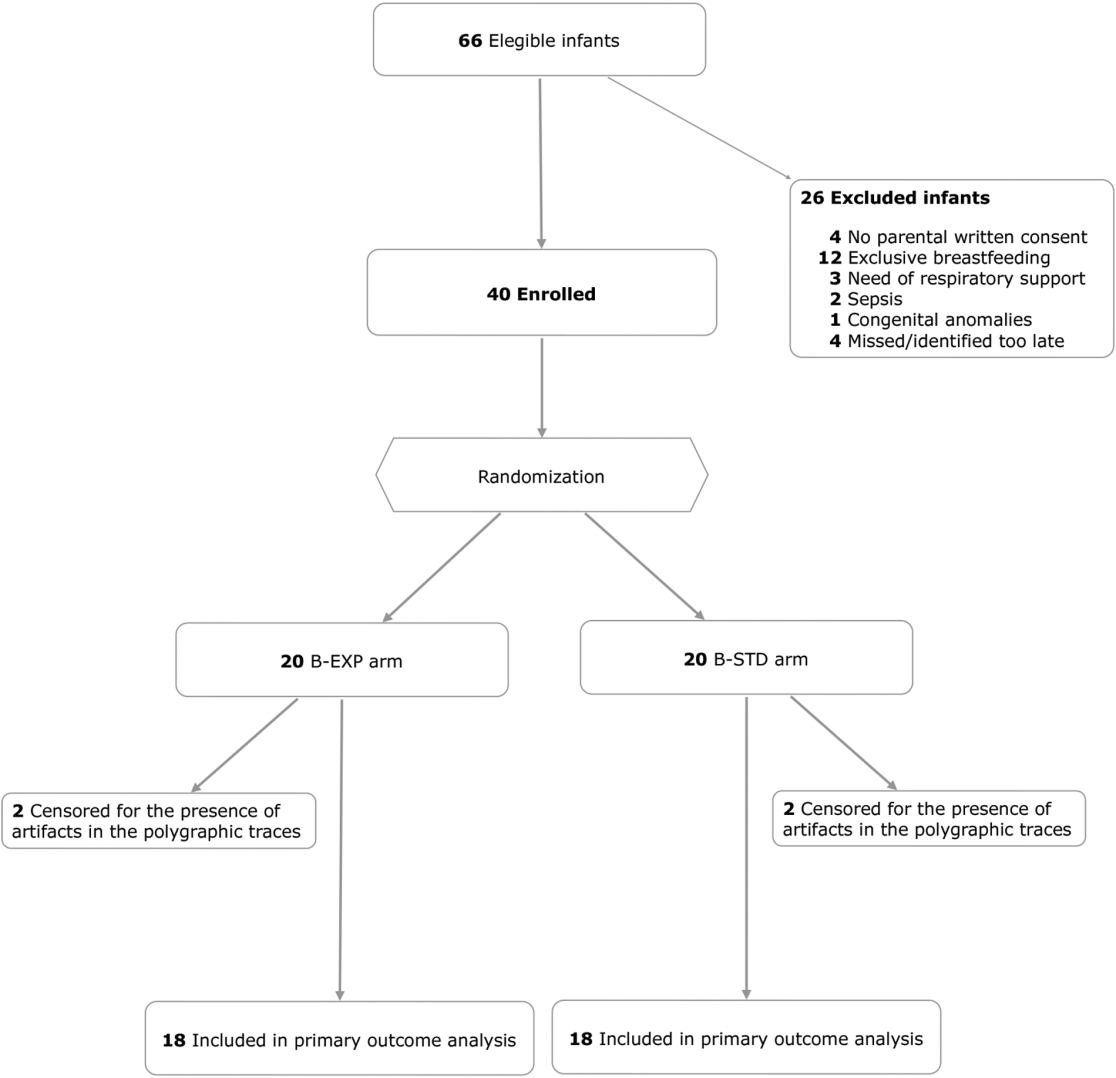
Study design and infants’ distribution. B-EXP, valved infant-bottle with ergonomic teat; B-STD, standard infant-bottle.

Four infants (2 B-EXP; 2 B-STD) were censored for the presence of artifacts in the polygraphic traces. Infants’ distribution through the study is shown in Figure 3. Baseline characteristics of the study population by arms are reported in Table 1.

**Table 1:** Characteristics of the study population by arm.

Baseline characteristics	B-EXP (*n* = 18)	B-STD (*n* = 18)	*P*
Birth weight, median (IQR), g	2,510 (2,290–2,610)	2,570 (2,435–2,630)	0.199
GA, median (IQR), weeks	35.00 (35.0–36.0)	35.50 (34.3–36.0)	0.931
Cesarean section, no. (%)	8 (44.4)	6 (30.0)	0.322
Female, no. (%)	9 (50)	11 (61)	0.738
SGA^a^, no. (%)	2 (11.1)	–	0.486
LGA^b^, no. (%)	–	–	–
IUGR, no. (%)	1 (5.5)	–	0.486
Apgar score at 5 min, median (IQR)	8 (8–9)	8 (8–9)	0.452
Age at monitoring, median (IQR), days	2.00 (1.00–2.75)	2.00 (1.00–2.00)	0.484
PMA at monitoring, median (IQR), weeks	35.30 (35.2–36.3)	35.70 (35.3–36.3)	0.994
Weight at monitoring, median (IQR), g	2,340 (2,175–2,522)	2,445 (2,402–2,460)	0.274

IQR, interquartile range; GA, gestational age; SGA small for gestational age; LGA, large for gestational age; IUGR, intrauterine growth restriction; PMA, postmenstrual age.

^a^ SGA: birthweight <10th centile according to INeS charts (10).

^b^ LGA: birthweight >90th centile according to INeS charts (10).

### Primary outcomes

The median swallowing/breathing ratio was 1.11 (1.03–1.23) in the B-EXP arm and 1.75 (1.21–2.06) in the B-STD arm (*p* = .003).

### Secondary outcomes

In the B-EXP arm the meal duration and the effective extraction time were longer than in the B-STD arm. In addition, a lower swallowing events frequency and a lower apnoea events frequency were observed in the B-EXP arm. Detailed secondary outcomes results are shown in Table 2.

**Table 2:** Feeding characteristics of the study population by arm.

	B-EXP (*n* = 18)	B-STD (*n* = 18)	*p*
Meal characteristics	Median (IQR)	Median (IQR)	
Meal volume (ml)	20.00 (15.00–27.50)	20.00 (15.00–20.00)	0.279
Total meal duration (sec)	255.00 (165.00–390.00)	184.00 (101.75–215.50)	**0**.**018**
Number of interruptions (*n*)	1.5 (1.00–3.00)	2.0 (0.25–4.75)	0.412
Total duration of interruptions (sec)	83.00 (32.75–147.75)	59.00 (4.50–132.00)	0.585
Effective extraction time (sec)	140.00 (98.00–274.00)	94.85 (43.25–136.00)	**0**.**026**
Milk flow (ml/min)	8.88 (5.32–12.17)	9.64 (5.52–25.54)	0.223
Recorded events	Median (IQR)	Median (IQR)	
Sucking events (*n*)	207.50 (148.00–292.00)	154.50 (107.50–368.00)	0.569
Swallowing events (*n*)	123.00 (87.50–196.50)	91.00 (68.75–150.00)	0.179
Breathing events (*n*)	98.00 (75.25–194.25)	58.00 (30.50–92.75)	**0**.**005**
Apnea events (*n*)	1.00 (1.00–2.00)	2.00 (1.00–3.75)	0.206
Events frequency^a^	Median (IQR)	Median (IQR)	** **
Sucking frequency (*n*/min)	71.70 (60.61–96.99)	93.32 (85.22–112.50)	**0**.**034**
Swallowing frequency (*n*/min)	51.98 (43.34–57.08)	57.64 (51.87–65.36)	**0**.**040**
Breathing frequency (*n*/min)	46.03 (42.20–47.90)	43.80 (29.43–48.08)	0.146
Apnea frequency (*n*/min)	0.43 (0.22–1.20)	1.00 (0.55–2.49)	**0**.**049**
Suck-swallow-breath coordination	Median (IQR)	Median (IQR)	** **
Sucking/swallowing rate (*n*)	1.50 (1.31–1.66)	1.84 (1.50–3.21)	0.053
Sucking/breathing rate (*n*)	1.61 (1.29–2.36)	2.59 (2.25–3.87)	**0**.**006**
Swallowing/breathing rate (*n*)	1.11 (1.03–1.23)	1.75 (1.21–2.06)	**0**.**003**

^a^ The event frequencies were calculated considering the extraction time.

Statistically significant *p*-values are indicated in bold type.

No differences were found in the median blood oxygen saturation and heart rate in the two arms. At discharge breastfed infants were 13 (61.9%) in the B-EXP arm and 8 (44.4%) in the B-STD arm (*p* = 0.091).

**Figure 4:**
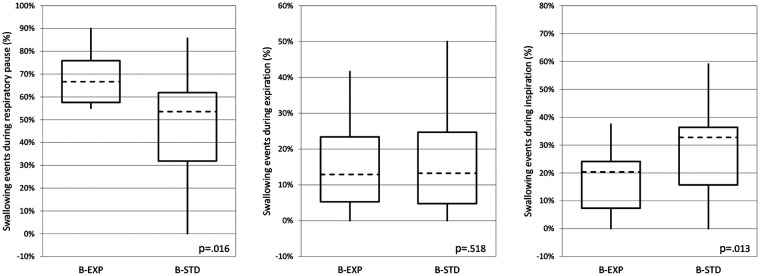
Swallowing events distribution during respiratory phases. B-EXP, valved infant-bottle with ergonomic teat; B-STD, standard infant-bottle. The dashed horizontal line, boxes, and whiskers represent median, IQR, and minimum-maximum, respectively. The differences were evaluated with the Mann Whitney test.

Finally, considering swallowing events classified by the respiratory phase in which they occurred, the frequency of P-Sw events was higher while the frequency of I-Sw events was significantly lower in the B-EXP arm compared to the B-EXP arm. No differences in E-Sw events frequency were observed between the two arms. (Figure 4).

## Discussion

In the early days of life, infants, particularly if preterm, encounter challenges with oral feeding due to their developmental immaturity and lack of experience (12). Newborn infants within 48 h of life have a stable and uniform swallow rate, but their breathing is not stably coordinated with swallowing, and ventilation is reduced during feeding especially if they are bottle-fed (5, 6).

Our starting hypothesis was that a feeding system based on a valved bottle could promote a more physiological coordination of swallow and breathing, preventing milk drip and eliminating internal vacuum resistance, making feeding more efficient and adapted to infants oral skill level (13).

To the best of our knowledge, this is the first RCT evaluating the effect of a feeding system based on a valved infant-bottle with an ergonomic teat compared to a standard infant-bottle on the coordination of sucking-swallowing-breathing patterns in a group of late preterm infants.

For a specific and unbiased evaluation, we concurrently recorded cardiorespiratory activity and sucking, swallowing, and breathing events during a meal using the synchronized polygraphy. This approach enabled us to accurately measure the frequency of each event and analyze the temporal correlations between them (1).

Our primary outcome was the swallowing/breathing ratio. The literature describes this parameter as strongly associated to the degree of maturity of the sucking-swallowing-breathing pattern expressed at its highest level by a swallowing/breathing ratio of 1:1 (8, 14, 15). This ratio is typical of term breastfed infants and is considered the optimum pattern for a physiological and safe feeding (6).

Our results showed that infants in the B-EXP arm had a lower swallowing/breathing ratio, closer to the ideal ratio of 1:1, compared to infants in the B-STD arm.

This effect had an important impact on the quality of breathing during swallowing by reducing apnoea frequency by about 50% in the B-EXP arm.

The feeding device used in the B-EXP arm featured two additional characteristics compared to the feeding device in the B-STD arm, designed to work synergistically. These included an ergonomic teat designed to simulate breastfeeding and facilitate perfect lip adherence to the teat, as well as a valve system to enable the infant to regulate milk extraction synchronously with their own swallows. Unlike standard infant-bottles, which release a continuous flow of milk due to the force of gravity and regulated by the tilt of the bottle, the viscosity of the milk, and the shape and the size of the exit hole, the B-EXP feeding device produce an intermittent flow controlled by the infant's activity. The infant's sucking creates a vacuum, which activates the valve and controls the milk's release making bottle feeding more similar to breastfeeding.

It is plausible that the use of the B-EXP replicates suction and breathing patterns more similar to those observed during breastfeeding, thereby facilitating the central nervous system in synchronizing swallows and breaths more effectively. This could result in a more continuous and safe feeding experience, allowing neonates to more regularly alternate between swallows and breaths (4).

Additionally, it should be noted that the valve system in the B-EXP feeding device allows air to enter the bottle with each suckling action, thereby equalizing the internal pressure in the bottle. Conversely, as evidenced in prior research, the B-SDT feeding device is characterized by a gradual build-up of negative pressure within the bottle during the sucking burst, which could potentially hamper the feeding efficiency of the infant (16).

Infants in the B-EXP arm exhibited an overall longer duration of feeding, primarily due to an extension in the actual extraction time. This outcome could be attributed to the different feeding patterns observed in the two groups: infants in the B-STD arm often had more sucks per swallow and more swallows per breath. This feeding pattern allows for a reduced extraction time but necessitates apnoeic swallows, leading to fatigue and an increased risk of inhalation. In contrast, infants in the B-EXP arm display longer extraction times during which they breathe more consistently and regularly between swallows. Similar results were observed in breastfed infants in studies comparing breastfeeding methods to bottle feeding (17, 18), reinforcing the hypothesis that infant-bottles equipped with valves contributes to the development of a more coordinated and physiological feeding pattern.

The research methodology employed facilitated a detailed analysis of the temporal associations between swallowing and respiratory phases, distinguishing swallowing occurred during inspiration, expiration or a pause in breathing. Compared to the B-STD arm, the B-EXP arm demonstrated a significant decrease of I-Sw frequency and an increase in P-Sw frequency.

This finding is very important as swallowing that occurs during short respiratory pauses between the end of an expiration and the beginning of an inspiration (low lung volume), or between the end of an inspiration and the beginning of an expiration (high lung volume) are associated with no airflow and are defined as safe, whereas swallowing that occurs during the inspiratory phase exposes the infant to the greatest risk of inhalation (1).

E-Sw frequency was similar in the 2 arms. This coordination pattern is potentially the safest for airway protection and may facilitate laryngeal elevation and cricopharyngeal sphincter opening. It is more typical of adults and tends to be more represented in infants as they become more mature and experienced in oral feeding (19), regardless of the type of infant-bottle used.

This study suffers from certain limitations that must be considered: first, the inability to perform a blind comparison that would have made our observations more robust; second, the methodology used proved to be very sensitive, but produced many motion artifacts during the recordings, forcing us to exclude 10% of the enrolled infants from the analysis; Finally, the classification of the swallow-respiration relations we used was simplified compared to that used in previous physio pathological studies (1, 11, 20). This choice decreased the details considered, but allowed an easier reading of the traces, reducing conflicts of interpretation.

## Conclusions

Breastfeeding is highly recommended for late preterm infants since birth. Our research suggests that infant bottles with a valved system mimic the natural coordination of breastfeeding more closely than standard bottles. They also reduce apnea episodes and the risk of inhalation during feeding. When exclusive breastfeeding is not possible, valved infant bottles with an ergonomic teat promote improved coordination of sucking-swallowing-breathing, favoring a more mature feeding pattern. Further longitudinal studies with larger cohorts are required to evaluate the long-term effects of using these feeding devices.

## Data Availability

The raw data supporting the conclusions of this article will be made available by the authors, without undue reservation.
